# Computational characterization and analysis of molecular sequence data of *Elizabethkingia meningoseptica*

**DOI:** 10.1186/s13104-022-06011-5

**Published:** 2022-04-09

**Authors:** Neha Girdhar, Nilima Kumari, A. Krishnamachari

**Affiliations:** 1 Department of Bioscience and Biotechnology, Banasthali Vidyapith, Jaipur, 304022 Rajasthan India; 2 School of Computational and Integrative Sciences, Jawaharlal Nehru University, New Delhi, 110067 India

**Keywords:** Bioinformatics, *Elizabethkingia meningoseptica*, Genome annotation, Pathway analysis, Subtractive genomics

## Abstract

**Objective:**

*Elizabethkingia meningoseptica* is a multidrug resistance strain which primarily causes meningitis in neonates and immunocompromised patients. Being a nosocomial infection causing agent, less information is available in literature, specifically, about its genomic makeup and associated features. An attempt is made to study them through bioinformatics tools with respect to compositions, embedded periodicities, open reading frames, origin of replication, phylogeny, orthologous gene clusters analysis and pathways.

**Results:**

Complete DNA and protein sequence pertaining to *E. meningoseptica* were thoroughly analyzed as part of the study. *E. meningoseptica* G4076 genome showed 7593 ORFs it is GC rich. Fourier based analysis showed the presence of typical three base periodicity at the genome level. Putative origin of replication has been identified. Phylogenetically, *E. meningoseptica* is relatively closer to *E. anophelis* compared to other *Elizabethkingia* species. A total of 2606 COGs were shared by all five *Elizabethkingia* species. Out of 3391 annotated proteins, we could identify 18 unique ones involved in metabolic pathway of *E. meningoseptica* and this can be an initiation point for drug designing and development. Our study is novel in the aspect in characterizing and analyzing the whole genome data of *E. meningoseptica*.

**Supplementary Information:**

The online version contains supplementary material available at 10.1186/s13104-022-06011-5.

## Introduction

In 1959, Elizabeth O King, discovered *Elizabethkingia* (renamed in 2005) [1], earlier known as *Chryseobacterium*. It is a non-glucose fermenting, non-motile, catalase-oxidase positive gram negative bacteria belonging to *Flavobacteriaceae* family, ubiquitous in soil, fresh and salty water [2]. The genus comprises of six species [3] that is, *E. meningoseptica* associated with meningitis and sepsis in premature neonates, [4, 5] *E. anophelis* isolated from the midgut of *Anopheles gambiae* mosquitoes which causes respiratory tract illness in human [6], *E. miricola*, isolated from condensation water on the Mir space station of Russia collected in 1997 [7], and *E. brunniana*, *E. ursingii* and *E. occulta* (three CDC genomospecies) [8].

*Elizabethkingia meningoseptica* is causative agent of meningitis in neonates and sepsis in immunocompromised patients [9]. The occurrence of nosocomial infection has risen, mainly in patients, with prolonged hospitalization, treated with invasive procedures, subsequently on use of broad-spectrum antimicrobials as well as having concomitant infections [10]. The mortality rate in patients infected with *E. meningoseptica* is significantly higher due to its unusual resistance pattern and mechanism [11]. Further studies are needed to initiate the most effective therapeutic approach. One can follow the time consuming and labor-intensive experimental approach but advancement in bioinformatics field provided enormous software tools, that are used to analyze and extract information from the molecular sequence, structure, expression and pathway data [12, 13].

The current study focused on analyzing the whole genome data of *Elizabethkingia* to unravel the embedded features hitherto not reported, secondly to explore the possibility of getting some lead in the directions of possible novel therapeutic candidates. Accordingly, we have studied genomic features, origin of replication sites, phylogenetic relationships, comparative genomics among *E. meningoseptica* species and further explored subtractive genomics approach together with pathway analysis.

## Main text

### Methods

#### Genome analysis of *E. meningoseptica* G4076 and its comparisons with *Elizabethkingia* family

The whole genome (Accession Number NZ_CP016376) and protein sequences of *Elizabethkingia meningoseptica* G4076 were downloaded from NCBI (www.ncbi.nlm.nih.gov). Nucleotide composition of genome was obtained using ORIS software [14]. To find all open reading frames in the genome, ORF finder, a graphical tool was used (https://www.ncbi.nlm.nih.gov/orffinder/) [15]. CG-Viewer was used for plotting circular plot of genomes [16]. Discrete Fourier Transform based computational approach using customized python codes was carried out to see the typical three-base periodicity feature embedded in *E. meningoseptica* genomic sequence [17]. Rapid Annotation using Subsystem Technology (RAST) server was carried out for studying genome annotation [18, 19]. Ori-Finder [20] and ORISv1.0 [14] software tools were used to identify putative origin of replication (oriC) sites in the genome. MegaX software was utilized to carry out phylogenetic analysis for species within the same genus such as *E. miricola*, *E. meningoseptica*, *E. anophelis*, *E. bruuniana*, *E. ursingii* and *E. occulta* as well as *Flavobacterium coloumnare* ATCC49512, *Riemerella anatipestifer* ATCC11845 (other genus in same family) [21]. The orthologous gene identification among *Elizabethkingia* species was carried out using Orthovenn2 with default parameters [22, 23].

#### Subtractive genomics based computational analysis

All protein sequences of *Elizabethkingia meningoseptica* G4076 and *Homo sapiens* (Host) were downloaded from NCBI database [24, 25]. Out of the total 3406 proteins in *E. meningoseptica*, hypothetical proteins and proteins having length less than 100 amino acids were discarded. Remaining 2503 proteins were subjected to BLASTP against proteomes of *Homo sapiens* [26]. Based on previous studies, expectation value cut off of 10^–4^ and minimum bit score of 100 used as threshold to shortlist non-homologous proteins [27]. Further, these non-homologous proteins were queried against Database of Essential Genes (DEG) server to get a list of essential genes for *E. meningoseptica* using e-value cut off 10^–10^ and bit score value of 100 as threshold [28]. These shortlisted essential genes that were non-homologous to host and essential for bacteria were studied further with respect to metabolic pathway.

#### Metabolic pathway analysis and subcellular localization prediction

Essential non-homologous proteins of *E. meningoseptica*, were further analyzed using KAAS (KEGG Automated Annotation Server) in order to study metabolic pathways [29]. KEGG analysis performed BLAST comparison against available KEGG gene database and provide metabolic pathway maps including KO and EC number for a particular gene. To determine the location of proteins in a cell PSORTb version 3.0 server was used [30]. The essential gene subjected to BLASTP analysis against FDA approved drug targets from Drugbank to search novel drug targets. Targets with identification of more or equal 80% are druggable targets and others that show considerable low degree of matching with already approved drug target can be used as novel targets for new drug identification [31].

### Results

#### Genomic features of *E. meningoseptica* G4076 and its comparison with other species

The whole genome data of *E. meningoseptica* G4076 having length of 3,873,125 bp showed a mean GC content of 36.5%, number of genes as per annotation is 3477 and the percentage base composition viz %A ≈ %T i.e., 31.76 and %G ≈ %C i.e., 18.23 calculated using ORIS software [14] (Additional file 1: Figure S1) which is in agreement with Chargaff’s parity rule [32]. Open reading frame is effective in identifying genes that encodes proteins. Total number of 7593 ORFs were found in whole genome. The products are of varying length and it shows that the number of ORFs found are actually slightly more than the annotated number of proteins (Additional file 1: Figure S2). To visualize sequence conservation, the circular genome plot was created using CG view Server (Additional file 1: Figure S3). Gene coding segments of *E. meningoseptica* genome does show the typical three-base periodicity indicating underlying codon structure that enables us to predict and identify all possible genes in majority of the bacterial genome with very high accuracy [17]. Additional file 1: Figure S4A shows all the bases considered for the fourier spectrum and indicates the presence of three base periodic signal as seen in most of bacterial genomes. Signal strength is prominent for purine-pyrimidine (Additional file 1: Figure S4B) whereas in the case of individual bases it is considerably low (Additional file 1: Figure S4C–F).

RAST server shows annotated data indicating 3477 putative genes, 61 RNAs which includes 4,4,4 (5S, 16S, 23S) ribosomal RNAs and 49 tRNAs and 335 subsystems (set of functional role) under 27 categories [18]. Sixty two coding sequences were related with antibiotics resistance and toxic compounds which suggests *E. meningoseptica* might be multiple drug resistant (Additional file 1: Figure S5).

**Fig. 1:**
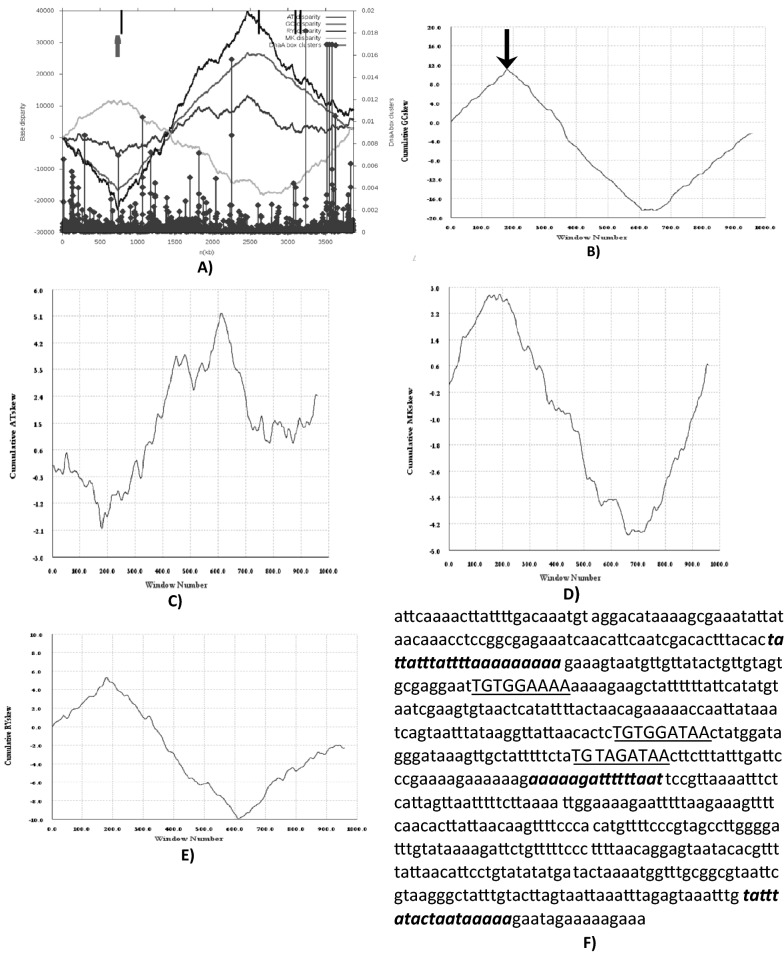
Output results using Ori-Finder—**A** Z curve (AT, GC, RY and MK disparity) for *Elizabethkingia meningoseptica* G4076. Peaks with the diamonds show DnaA boxes, bold arrow indicates oriC location, and solid short black lines show replication marker genes i.e., dnaN, dnaA, gidA, hemE etc). **B**–**E** Cumulative GC, AT, MK, RY skew graph of *E. meningoseptica* G4076 using ORISv1.0 software tool having window size 40,000 with increment of 4000 bp. Bold solid arrow indicates putative ori site. **F** OriC sequence wherein showed DnaA boxes (capitalized and underlined) with not more than one mismatch to *E. coli* DnaA box. AT clusters, in oriC region are shown in bold

Ori-Finder (a web based software tool for finding oriCs) predicted oriC region of 649 bp ranging from 740,720 bp to 741,368 bp having three DnaA box sequence motifs (TTATCCACA) with no more than one mismatch. Further, replication related gene, dnaA located from 2,613,273 to 2,614,727 bp which is followed by dnaN gene (Fig. 1A) [20]. A cluster of three DnaA boxes and two AT rich DNA unwinding elements (DUE) are indication of functional chromosomal origin (Fig. 1F). Similar kind of result was found with ORIS v1.0 software tool. DNA asymmetry, distribution of DnaA boxes as well as location of the dnaA gene help in predicting *OriC* regions [33–36]. Both graphs enable us to pin-point or identify ORI/TER site. The difference in the position (genome coordinates) of OriC predicted by Ori-Finder and ORIS are well within 1 kb and hence, close agreement.

Genomic comparison among *Elizabetkingia* species [*E. meningoseptica* G4076 (WP_016198861.1), *E. miricola* BM10 (WP_034866598.1), *E. ursingii* G4123 (WP_078402796.1), *E. anopheles* NUHP1(WP_009086312.1) *E. bruuniana* G0146(WP_034866598.1), *F. columnare* ATCC49512(WP_014166114.1), *R. anatipestifer* ATCC11845(WP_004918717.1)] has been done using MEGAX software. It depicts phylogenetic relatedness by comparing homology of protein sequence specifically 16S rRNA processing Protein RimM (Ribosomal maturation factor RimM) (Additional file 1: Figure S6) [37]. It has been found that *E. meningoseptica* are relatively at a large phylogenetically distance from other species of *Elizabethkingia*. Cluster of orthologous gene analysis of *E. meningoseptica* G4076 was compared with four other species of *Elizabethkingia* to provide insights into biological process, molecular functions and cellular components [22, 23]. It was found that among 3970 clusters, 1401 were orthologous clusters which contain at least two species and 2569 singletons. The number of orthologous genes shared by five species of *Elizabethkingia* genome was 2606 whereas 17 COGs were present only in *Elizabethkingia meningoseptica* G4076 genome which is involved only in metallopeptidase activity (Additional file 1: Figure S7). In pairwise comparison ranges varies from 3396 to 3409 COGs (Additional file 1: Figure S7C).

#### Prediction of essential genes in *Elizabethkingia meningoseptica*

**Fig. 2:**
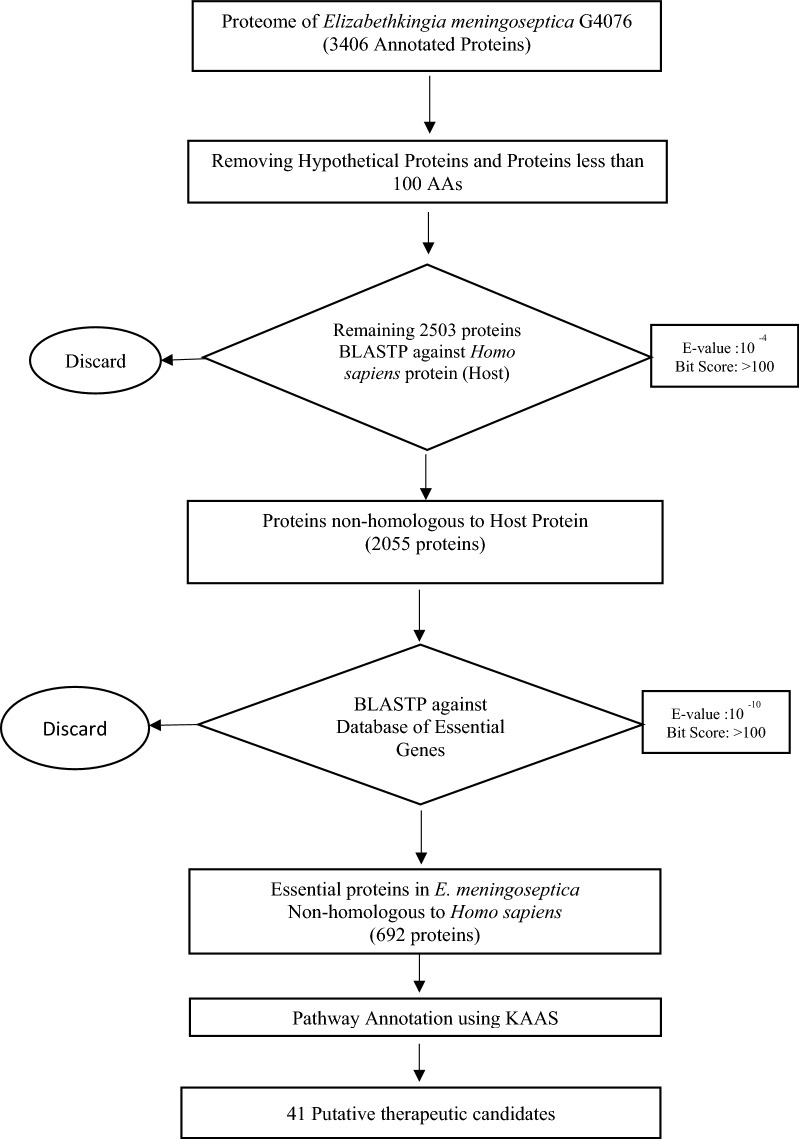
Flow chart for identification of putative drug targets

Subtractive genomic analysis is unique, fast and efficient method for identifying essential genes in pathogenic species that are non-homologous to human (host). These non-homologous essential genes can be used as putative drug targets against pathogens [38]. The genome of *E. meningoseptica* G4076 has 3391 annotated proteins. After exclusion of protein which are < 100 amino acids and hypothetical, remaining 2503 were subjected to BLASTP against proteins of *Homo sapiens* (host). Using e-value cut off 10^–4^ and bit score > 100, it was found total of 2052 proteins were non-homologous to host protein. Thereafter, these proteins were subjected to BLAST analysis using DEG server and using e-value cut off 10^–10^ and bit score > 100, shortlisted 692 proteins that are essential for *E. meningoseptica* G4076 but absent in host (Additional file 1: Table S1). DEG contains gene that plays important role in cell survival and can be novel targets for antibacterial drugs (Fig. 2).

#### Metabolic pathway analysis of essential gene and subcellular localization prediction

The shortlisted non-homologous essential genes were analyzed using KEGG database for metabolic pathway annotation. It was found, only 41 out of 692, are present in pathogen as unique pathways (Table 1). Majority of them were involved in DNA binding response regulator, ribosomal proteins, replication and repair, Glycan biosynthesis, protein folding and sorting, two-component system, biotin metabolism and ATP transporters. It is very important for drug designing to determine whether target protein resides on cell surface or in cytoplasm. Localization of proteins play important role in drug binding and action. Subcellular localization reveals, out of 41 target proteins, 80% of total are cytoplasmic, rest located in periplasm or cytoplasmic membrane and no extracellularly proteins were obtained (Additional file 1: Figure S8). Extracellularly secreted proteins may be better opted for vaccine development. Here, it is clear that majority of proteins resides in cytoplasm and cytoplasmic membrane that further can be considered as potential therapeutic targets. Unique *E. meningoseptica* essential proteins non-homologous to host further subjected to BLASTP against FDA approved drug targets from Drugbank which shortlisted to 18 target proteins. Out of which penicillin binding protein (2), ABC transporter ATP binding proteins (2) that targets for broad-spectrum antibiotics. The rest includes ribosomal proteins (rpsB, rpsl, rpsG, rpsJ, rpsE, rpsM, rpsK, rpsD, rplD, rplP), recombination protein (recR), DNA polymerase subunit III tau (dnaX), and signal peptidase which could be further explored as starting point for discovering novel drug candidate. Ribosomal proteins can be more suitable candidates for drug binding as it mainly involves in translation. Another work also lend support for choosing the specific drug target [39]. In that regard, computational analysis may include homology modelling and docking of selected candidate.

**Table 1:** Unique and novel essential genes in *E. meningoseptica* G4076

Protein product	Protein name	KEGG orthology	EC	Subcellular localization
Two component system				
WP_016198590.1	Response regulator transcription factor	K07665	–	Cytoplasmic
WP_016199055.1	LytTR family DNA-binding domain-containing protein	K07705	–	Cytoplasmic
WP_016198441.1	Response regulator transcription factor	K07665	–	Cytoplasmic
WP_016199099.1	LytTR family DNA-binding domain-containing protein	K07705	–	Cytoplasmic
WP_016200043.1	VanW family protein	K18346	–	Unknown
WP_016199769.1	Two-component sensor histidine kinase	K07636	2.7.13.3	Cytoplasmic membrane
Beta-lactam resistance				
WP_016170088.1	Penicillin-binding protein 2^a^	K05515	3.4.16.4	Cytoplasmic membrane
WP_016170028.1	Transglycosylase domain-containing protein^a^	K05366	2.4.1.129	Cytoplasmic membrane
DNA replication				
WP_016169884.1	Ribonuclease HI	K03469	3.1.26.4	Unknown
WP_016199473.1	DNA primase	K02316	2.7.7.101	Cytoplasmic
WP_019051072.1	DNA polymerase III subunit gamma/tau^a^	K02343	2.7.7.7	Cytoplasmic
WP_016198779.1	DNA polymerase III subunit delta\′	K02341	2.7.7.7	Cytoplasmic
Homologous recombination				
WP_016199810.1	Holliday junction branch migration protein RuvA	K03550	3.6.4.12	Cytoplasmic
WP_016198560.1	Holliday junction branch migration DNA helicase RuvB	K03551	3.6.4.12	Cytoplasmic
WP_016200627.1	Recombination protein RecR	K06187	–	Cytoplasmic
WP_016199534.1	DNA replication and repair protein RecF	K03629	–	
Translation				
WP_009085459.1	30S ribosomal protein S2^a^	K02967	–	Cytoplasmic
WP_016200426.1	30S ribosomal protein S9^a^	K02996	–	Cytoplasmic
WP_009087380.1	30S ribosomal protein S12^a^	K02946	–	Cytoplasmic
WP_009087378.1	30S ribosomal protein S7^a^	K02992	–	Cytoplasmic
WP_016197802.1	30S ribosomal protein S10^a^	K02946	–	Cytoplasmic
WP_009087341.1	50S ribosomal protein L4^a^	K02926	–	Cytoplasmic
WP_016197785.1	50S ribosomal protein L16^a^	K02878	–	Cytoplasmic
WP_009087327.1	50S ribosomal protein L14	K02874	–	Cytoplasmic
WP_016197784.1	50S ribosomal protein L24	K02895	–	Cytoplasmic
WP_009087314.1	30S ribosomal protein S5^a^	K02988	–	Cytoplasmic
WP_016197779.1	50S ribosomal protein L15	K02876	–	Cytoplasmic
WP_016197776.1	30S ribosomal protein S13^a^	K02952	–	Cytoplasmic
WP_009087288.1	30S ribosomal protein S11^a^	K02948	–	Cytoplasmic
WP_016170211.1	30S ribosomal protein S4^a^	K02986	–	Cytoplasmic
WP_016170209.1	50S ribosomal protein L17	K02879	–	Cytoplasmic
WP_016198862.1	30S ribosomal protein S16	K02959	–	Cytoplasmic
WP_016200457.1	50S ribosomal protein L20	K02887	–	Cytoplasmic
WP_016199408.1	30S ribosomal protein S1	K02945	–	Cytoplasmic
WP_016200561.1	50S ribosomal protein L9	K02939	–	Cytoplasmic
ABC Transporters				
WP_016198610.1	ATP-binding cassette domain-containing protein^a^	K09812	–	Cytoplasmic membrane
WP_016198126.1	ABC transporter ATP-binding protein^a^	K09810	7.6.2.-	Cytoplasmic membrane
Protein export				
WP_026149261.1	Signal peptidase I^a^	K03100	3.4.21.89	Cytoplasmic membrane
Methane metabolism				
WP_016198134.1	Phosphoenolpyruvate carboxylase^a^	K01595	4.1.1.31	Cytoplasmic
Base excision repair				
WP_016170024.1	Endonuclease III	K10773	4.2.99.18	Cytoplasmic
Biotin metabolism				
WP_016199146.1	Dethiobiotin synthase	K01935	6.3.3.3	Cytoplasmic

^a^Potential therapeutic candidates as per FDA approved drugbank

### Discussions

Meningitis and sepsis is a major illness in newborn and immunocompromised patients caused by *Elizabethkingia meningoseptica*. Though typical clinical diagnostics are used to identify the illness but a greater understanding of molecular based diagnosis is desired and it is a long term goal. Increase in number of cases in Intensive care units (ICUs) makes it big challenge for clinicians to deal and manage. In this context, comprehensive analysis of whole genome data and pathway analysis were explored as we do not see much work related to computational analysis. Accordingly, bioinformatics approach was undertaken for characterizing molecular sequence data of *Elizabethkingia*. Our study identified 41 unique proteins in *Elizabethkingia* with respect to the host using subtractive genomics which further narrow down to18 therapeutic target proteins using *in-silico* comparative genomics. The suitable shortlisted ribosomal proteins which are linked to translation may be useful for future treatment and management of the infection. We have studied in an integrated fashion of considering and analyzing sequence data of *E. meningosptica* together with pathway analysis. Our study is small step in the direction of rapid diagnosis and possible drug development.

## Limitations

The current investigation is limited to in silico study only.

## Supplementary Information

**Additional file 1: Figure S1.** Percentile distribution of DNA base composition in *E. meningoseptica* G4076 genome. **Figure S2.** Open reading Frame viewer—a Window showing ORFs on the interval from 1 to 50,000 nucleotides. **Figure S3.** Circular genomic plot of *E. meningoseptica*. **Figure S4.** Fourier Transform Spectrum. **Figure S5.** Annotation of *Elizabethkingia meningoseptica* G4076 genome using RAST server. **Figure S6.** Phylogeny tree of *Elizabethkingia* species. **Figure S7.** Cluster of genes, Venn diagram and pairwise heat map among *Elizabethkingia* species. **Figure S8.** Pie-chart showing subcellular localization of proteins. **Table S1.** List showing subtractive genomic and metabolic pathway analysis result of *E. menigoseptica*.

## Data Availability

The whole genome sequence of *Elizabethkingia meningoseptica* G4076 having Accession Number NZ_CP016376 was downloaded from NCBI site https://www.ncbi.nlm.nih.gov/genome/14625?genome_assembly_id=309079. All the protein sequences (numbering 3406) available in FASTA format were used for BLASTP analysis against human dataset option. Selected protein sequences (described in material method section) were further used as input for subtractive genomic analysis.

## Abbreviations

*E*.: *Elizabethkingia*
ORF: Open reading frame
GC: Guanine cytosine
RAST: Rapid Annotations using Subsystems Technology
COGs: Cluster of groups
CDC: Centre of Disease Control
DNA: Deoxyribonucleic acid
NCBI: National Center for Biotechnology Information
AT: Adenine thymine
MK: Amino-keto
RY: Purine pyrimidine
ORIS: ORI search
OriC: Origin or replication C
MEGA: Molecular evolutionary genetic analysis
ATCC: American type culture collection
bp: Base pair
kb: Kilobase pair
RNA: Ribonucleic acid
CDS: Coding sequences
DUE: DNA unwinding element
TER: Terminal
ICUs: Intensive Care Units
e-value: Expectation value
BLAST: Basic Local Alignment Search Tool
DEG: Database of essential genes
KEGG: Kyoto Encyclopedia of Genes and Genomes
FDA: Food and Drug Administration
