# Supplementary material for: Computational characterization and analysis of molecular sequence data of Elizabethkingia meningoseptica
Source: BMC Res Notes. 2022 Apr 9;15:133. doi: 10.1186/s13104-022-06011-5 (PMC8994065; doi:10.1186/s13104-022-06011-5)
Supplement: Supplementary file 1 — Additional file 1: Figure S1. Percentile distribution of DNA base composition in E. meningoseptica G4076 genome. Figure S2. Open reading Frame viewer—a Window showing ORFs on the interval from 1 to 50,000 nucleotides. Figure S3. Circular genomic plot of E. meningoseptica. Figure S4. Fourier Transform Spectrum. Figure S5. Annotation of Elizabethkingia meningoseptica G4076 genome using RAST server. Figure S6. Phylogeny tree of Elizabethkingia species. Figure S7. Cluster of genes, Venn diagram and pairwise heat map among Elizabethkingia species. Figure S8. Pie-chart showing subcellular localization of proteins. Table S1. List showing subtractive genomic and metabolic pathway analysis result of E. menigoseptica. [file 13104_2022_6011_MOESM1_ESM.docx]

**Supplementary Material**

**Figure S1**. Percentile distribution of DNA base composition in *E. meningoseptica* G4076 genome.


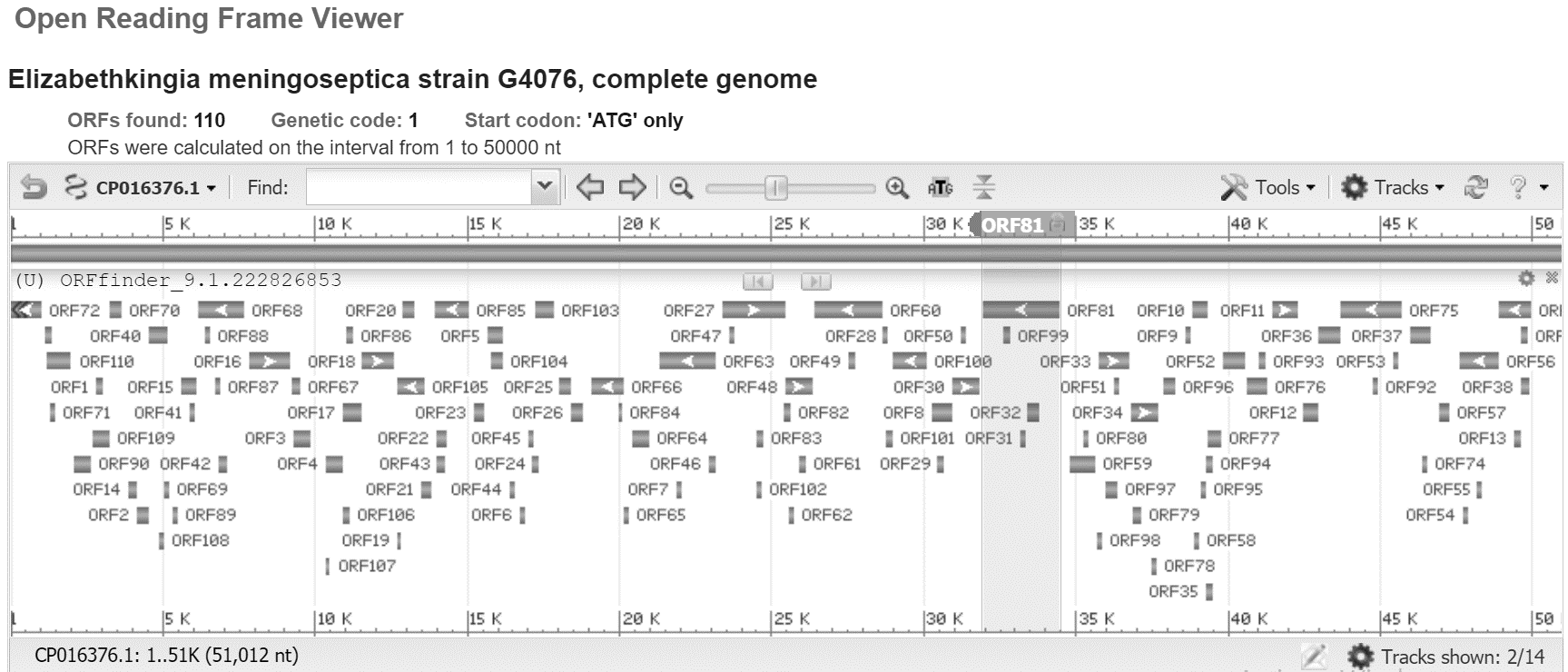


**Figure S2**. Open reading Frame viewer –a Window showing ORFs on the interval from 1 to 50000 nucleotides.


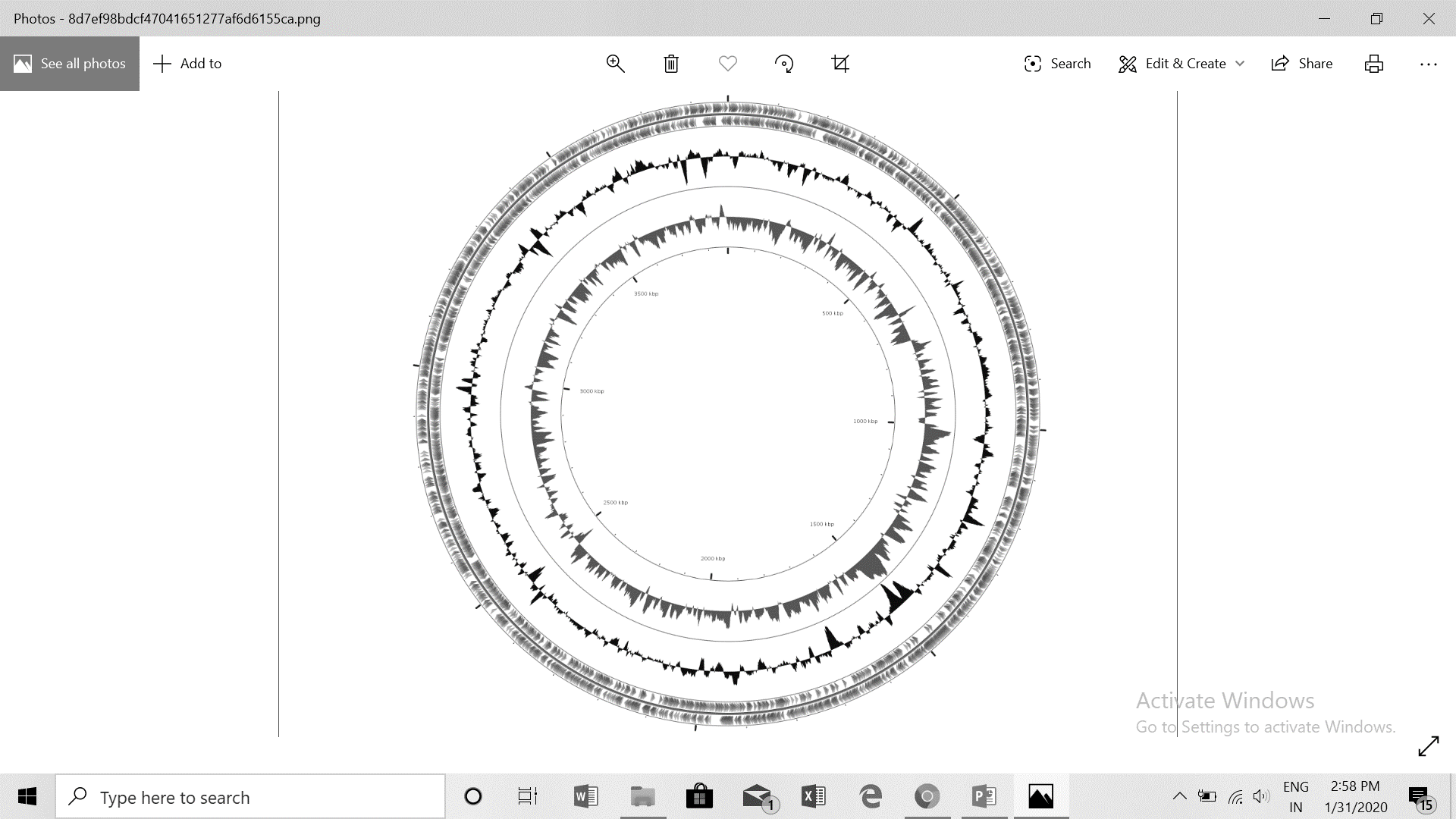


***E. meningoseptica* G4076**

**Figure S3.** Circular plot using CG View Server showed GC skew+ and GC skew- (inner circle) and GC content (middle circle). Outer circle corresponds to ORFs.


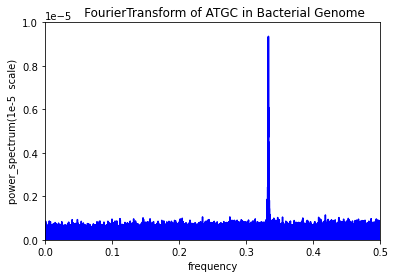

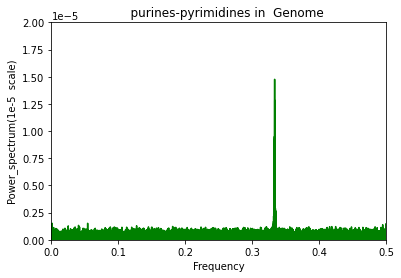

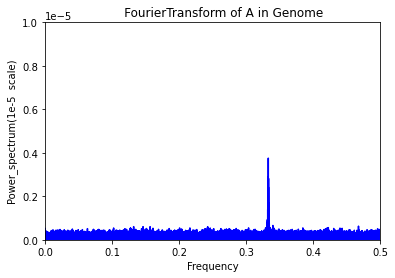

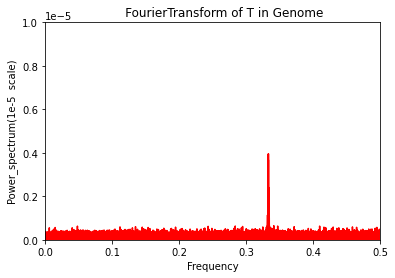

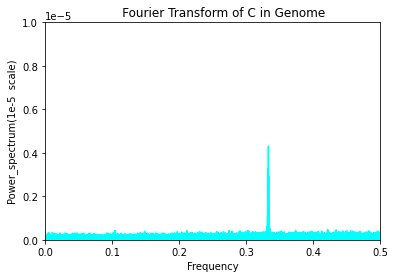

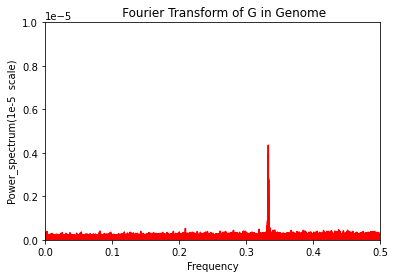


A

B

C

D

E

F

**Figure S4.** Fourier transform of *E. meningoseptica* genome.


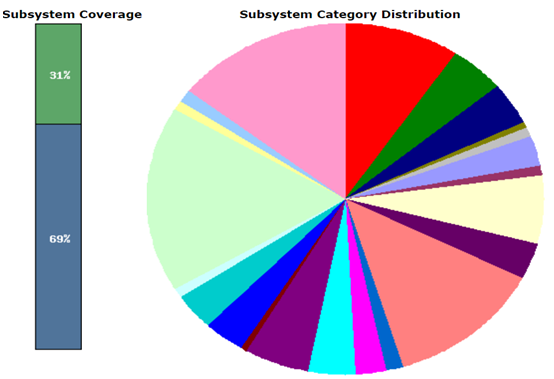

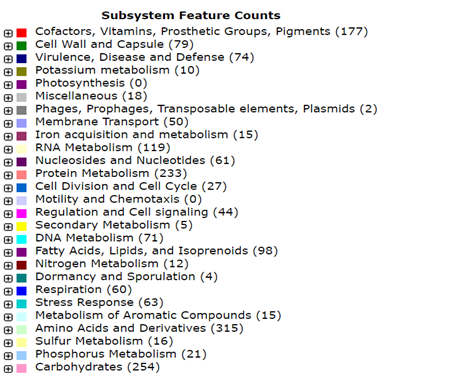


**Figure S5**. Genome of *Elizabethkingia meningoseptica* G4076 annotated using RAST server showing subsystems and their further 27 categories. Bar chart represents percentage of proteins included. Pie chart illustrated distribution of genes belong to various subsystem groups and their different categories.


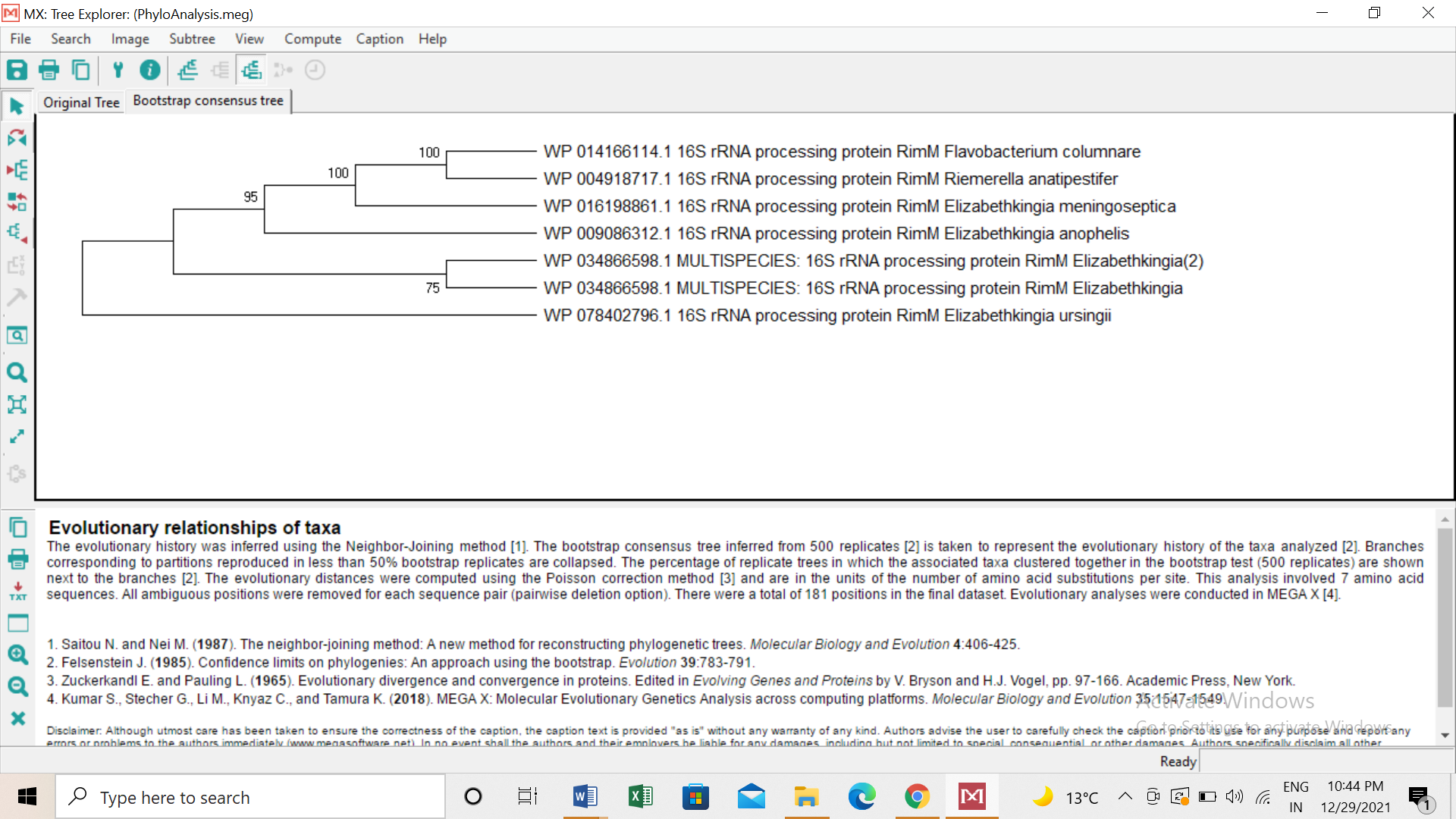


F**igure S6.** Phylogenetic tree was constructed using Neighbor-Joining method and analysis conducted by MEGA X.


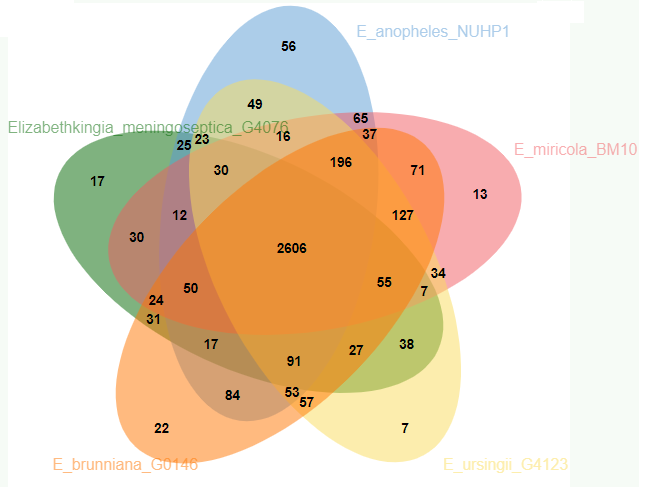

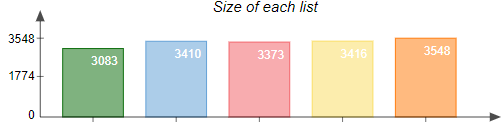


E.meningoseptica G4076

E. anophelis NUHP1

E. miricola BM10

E. ursingii G4123

E. brunniana G0146

**A)**


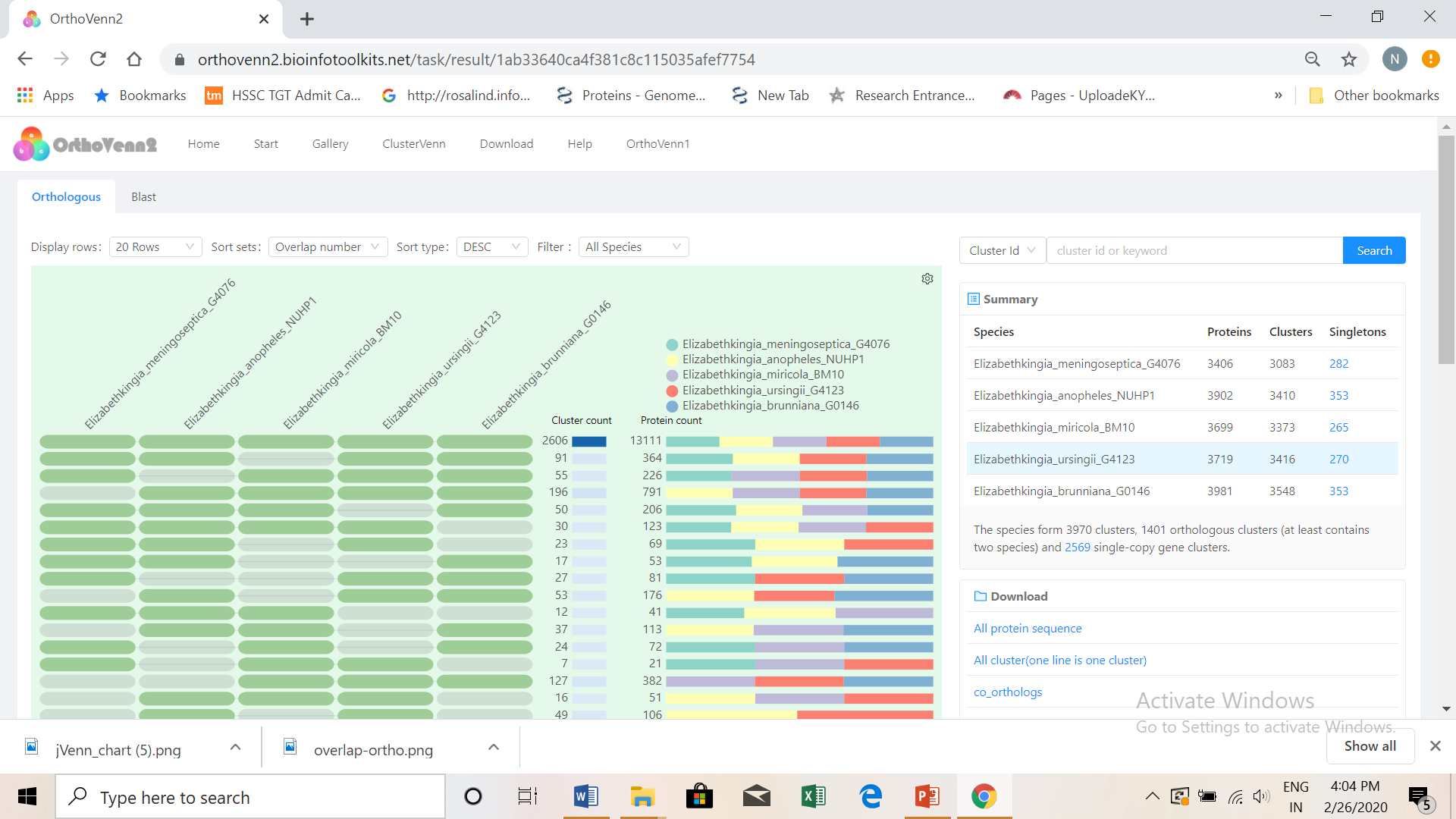


**B)**


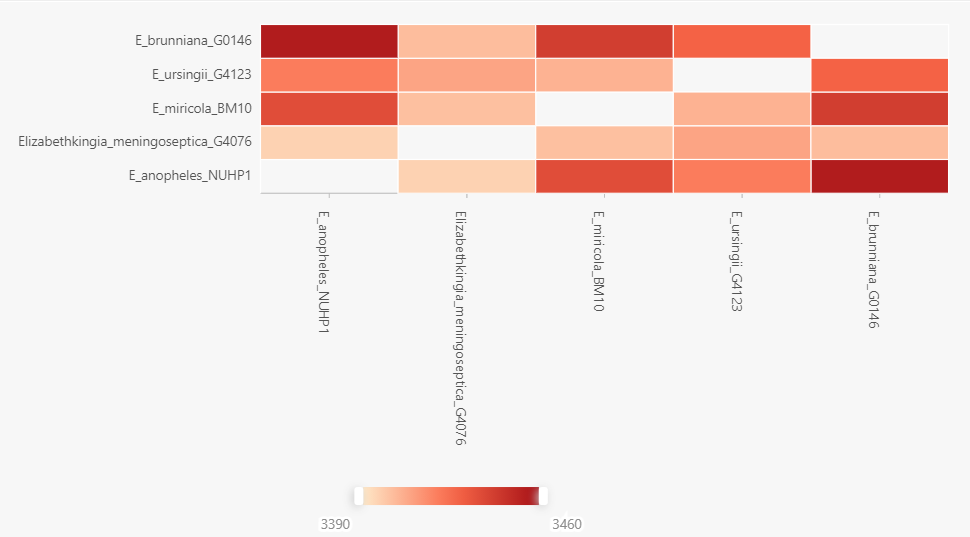


**C)**

**Figure S7. A)** Venn diagram showing the division of shared orthologous clusters in five species. **B)** Summary displaying Protein count, cluster count and singletons among five species. **C)** Representation of pairwise- heatmap of overlapping cluster number, wherein each cell defines the overlap cluster number between each pair of species.

**Figure S8.** Pie-chart representation of subcellular localization.

**Table S1.** List showing subtractive genomic and metabolic pathway analysis result of *E. menigoseptica*.

| **Features** | **Numbers** |
| --- | --- |
| Total number of proteins | 3406 |
| Hypothetical proteins | 794 |
| <100 amino acids | 94 |
| Nonhuman homologous proteins (e-value 10^-4^) | 1531 |
| Essential genes in DEG  (e-value 10^-10^) | 692 |
| Unique metabolic pathway for *E.meningoseptica* | 41 |
